# Habit

**Published:** 1875-07

**Authors:** B. Browne


### HABIT.

BY B. BROWNE.

No one who reads this article can deny the fact that all of us are but an aggregate of habits. We have our habit of dress, of speech, of walking, of standing. By them we are known more than anything else. They make for us our character by which men identify and distinguish us in our intercourse with the world.

It is a proverb that "habit is second nature." Looking into "Webster" for the definition of the word, we find, "habit, involuntary tendency to perform certain actions which is acquired by their frequent repetition." The word *custom* seems almost identical in meaning with *habit*, but yet bears a different relation—the first supposing an act of the will, and the other a law of our being, or "second nature." The expression, "the habit of devotion produces the custom of going to church," will illustrate this distinction in the use of the words.

At no period of life are any of us free from the influence of habits. From the moment a child exhibits the least independency of action, he commences to run counter to, or in harmony with the great ruling law of society, that we do not live for ourselves alone.

He who soonest recognizes this fact, and takes on those habits which do not bring him into antagonism with all who desire peace, and mutual concession of individual desire, will soonest obtain a good name in his circle of acquaintance.—Upon all sides silent influences are at work, which by a sort of attrition will wear against and wear off the points and angles of a thoroughly selfish, I may say natural nature.

The law of every one for himself, cannot find rule except by a despotic, compulsory yielding to every whim or caprice by those who have rights which are entitled to respect.

The first mentioned law of "living for others," will not give place to the latter of self first, always.

The infant who exacts homage from all the household, who humor his whims, pet his fancies, and finally builds up a petty monarchy, to render prompt obedience to its capricious temper, is forming a character which is conspicuous for only negative qualities. We see that this *character* is but the aggregation of a number of habits—a sum total belonging to him and distinguishing him for his life, except as they are given up or lost by after causes.

No one calls such a child lovely, and the less he is inflicted upon community the better for all.

The habit of thinking for and of others is formed in the infant over whom the loving but not overindulgent mother exhibits restraint before it can speak its wants. Learning to yield at this age, the influence of judicious government grows as readily as the contrary.

We see then, that this conformity of our desires to the established customs and habits of good society, is the object to be attained by the educators of our children. The power of habit is a subject for close study by every one. Many traits of character are called hereditary which are not so at all.—Two children of a family, who exhibit no distinguishing peculiarities, if separated and educated apart will give scarce a sign in their adult age of any relationship. Acted upon by diverse influences, their habits varied by their circumstances, their two characters are formed and they go out into the world to be estimated by the worth which society puts upon its members. The one shall be loved for sterling habits, and the other shunned for infamous ones.

There is not an assertion that I have made, which any one will deny. It is almost like a school boy's essay, to take up the subject and present the matter at all. This, however, is true of nearly everything which would be suitable for the pages of this journal. Because it is homely and worn thread-bare by scores of boys and girls in their "teens" to their t-y ty, there is much of proof that there is value in it. "What every one says must be true," is a wise saying. I will only ask the privilege of stirring up some possibly dormant mind to the consideration that he or she may have a habit or two which has become so much a part of his or her being as not to have offered any suggestion that it was not altogether lovely. Perhaps its owner would receive a better welcome in his or her circle of acquaintance if consideration of it should induce its advancement.

It is a good idea to occasionally step down and out of our circle and see just how we look. Treat ourselves with regard to characteristic adornments as we would in adorning our bodies. Let us get some friend to say whether the colors or fashion of our clothes please the mass of people, and let us ask the same or some other friend to pass a glass around us while we see ourselves just as others see us. Is not this or that habit foreign to the rest of our being, and if we sacrifice it altogether, will there not be a greater harmony in our appearance? We flatter ourselves usually to such an extent that we are unable to correctly estimate our character, hence we are liable to put on habits which we are in need to unload. Judicious friends can advise us, if we only show an inclination to profit by their judgments. Possibly we shall find some of our habits rather too old-fashioned to ensure our proper appreciation by others, yet we carry them about all unconsciously, or at the most, with an occasional wonderment why we attract attention where none is expected. It is a good idea to carry this analogy of habits in the sense of dress or clothing, with habits as defined by "Webster." Either one of them may bear criticism according to our customary use of them.

There is just one class of persons whom I wish to touch up, for they seldom awake to the consciousness that they are ever touched by the heaviest broadside of argument. If it does not mean you, you probably know some one whom it does mean, and you can go carry the news to them if you choose, thus making sure that some one has been hit. I refer to those who are drifting along, making no stir which will create a ripple in the perfect smoothness of their every day life. Good humored, blameless, they are actually uninteresting for that reason. They avoid independency in every thing, having a certain run of habits formed, till education stopped where the intellect seems to have been jammed, as it were, into a vise, and to have lost all power of free play. One is tempted to say—they be known for nothing worth being known for.

